# Serum Bilirubin Is Inversely Associated with Increased Arterial Stiffness in Men with Pre-Hypertension but Not Normotension

**DOI:** 10.1371/journal.pone.0146226

**Published:** 2016-01-12

**Authors:** Yao-Hsien Huang, Yi-Ching Yang, Feng-Hwa Lu, Zih-Jie Sun, Jin-Shang Wu, Chih-Jen Chang

**Affiliations:** 1 Department of Family Medicine, National Cheng Kung University Hospital, Tainan, Taiwan; 2 Department of Family Medicine, College of Medicine, National Cheng Kung University, Tainan, Taiwan; 3 Department of Family Medicine, National Cheng Kung University Hospital Dou-Liou Branch, Yunlin, Taiwan; Shanghai Institute of Hypertension, CHINA

## Abstract

**Objective:**

Serum bilirubin level has shown to be inversely associated with coronary atherosclerosis, and may serve as a protective biomarker of coronary artery disease. Serum bilirubin has also been shown to be negatively associated with brachial-ankle pulse wave velocity (baPWV) in men without a history of hypertension, and in men with hypertension. It is unknown whether such associations can be observed in the pre-hypertensive or normotensive population. This study thus aimed to investigate the relationship between serum bilirubin level and increased arterial stiffness in subjects with pre-hypertension and normotension for both genders.

**Methods:**

A cross-sectional sample of 3,399 apparently healthy subjects undergoing a medical check-up at National Cheng Kung University Hospital was enrolled between October 2006 and August 2009, after excluding subjects with serum total bilirubin level greater than 20.52 μmol/L. Increased arterial stiffness was defined as baPWV of 1,400 cm/s or higher as the dichotomous variable and bilirubin as the continuous variable.

**Results:**

Based on multiple linear regression analysis, serum bilirubin level was inversely associated with baPWV in non-hypertensive men (β = -0.066, p < 0.001) but not in non-hypertensive women. In addition, the inverse relationship between bilirubin level and baPWV was found statistically significant only in pre-hypertensive men (β = -0.110, p < 0.001). Multiple logistic regression analysis showed that serum bilirubin was inversely associated with increased arterial stiffness in men with pre-hypertension (odds ratio = 0.955, 95% confidence interval = 0.916–0.996, p < 0.05) but not normotension after adjustment for other confounding factors. However, the relationship between total bilirubin level and increased arterial stiffness did not reach statistical significance for female subjects with pre-hypertension and normotension.

**Conclusion:**

Serum bilirubin is inversely associated with increased arterial stiffness in men with pre-hypertension but not normotension. The association between bilirubin level and arterial stiffness was not found significant in women.

## Introduction

Arterial stiffness, which describes the rigidity of arterial walls and resulting loss of elasticity, is strongly associated with atherosclerosis at various sites in the vascular tree [1], and is known to be correlated with all-cause mortality and cardiovascular disease [2,3]. Among the noninvasive methods that can be used to quantify arterial stiffness, pulse wave velocity (PWV) is one of the most validated measures [4]. Previous research indicates that carotid-femoral pulse wave velocity (cfPWV) and brachial-ankle (baPWV) are indices of arterial stiffness that exhibit a similar extent of associations with cardiovascular disease risk factors and clinical events [5]. Although cfPWV is considered the gold standard of central arterial stiffness, it requires persistent lateral rotation of the patient’s neck, and exposure of the inguinal region. In contrast, baPWV is a more convenient measure, which only requires a pressure cuff wrapped over the limbs while the individual is in a supine position. In addition, recent prospective studies also suggest that baPWV is a significant risk factor for stroke and cardiovascular disease [6,7].

Bilirubin is the end product of heme catabolism in the systemic circulation [8]. Previous studies have suggested that serum bilirubin level is inversely associated with coronary atherosclerosis, and it may serve as a protective biomarker of coronary artery disease [9,10], which is possibly related to the potential antioxidant effect of bilirubin, although this negative correlation seems less clear in women [11,12]. Furthermore, Li et al. indicated that serum bilirubin was negatively associated with baPWV in men without history of hypertension [13], and Zhang et al. further demonstrated an independent inverse relationship between bilirubin and baPWV in hypertensive men [14]. However, it is unknown whether their association can be observed in pre-hypertensive men and even women. This study thus aimed to investigate the relationship between serum bilirubin level and increased arterial stiffness, as shown by baPWV ≥ 1,400 cm/s [15], in subjects with pre-hypertension and normotension for both genders.

## Methods

### Study Subjects

This study enrolled 7,559 subjects who had a medical check-up at National Cheng Kung University Hospital between October 2006 and August 2009. Based on a secondary data analysis without personal identification information, the requirement for informed consent was waived and this study was approved by the Institutional Review Board of National Cheng Kung University Hospital (B-ER-101-331).

We excluded individuals aged under 18, and those with a history of coronary heart disease, stroke, diabetes mellitus, hypertension and cancer, anemia (hemoglobin less than 135 g/L in men and less than 116 g/L in women), chronic kidney disease (estimated glomerular filtration rate < 60 ml/min or proteinuria), or taking medications known to influence blood pressure, plasma glucose and lipid profile. Subjects with an ankle-brachial index (ABI) less than 0.9 were excluded to rule out peripheral arterial occlusion [16]. Participants who had positive hepatitis B surface antigen, positive hepatitis C antibody, aspartate aminotransferase (AST) greater than 40 U/L, alanine aminotransferase (ALT) greater than 55 U/L and serum total bilirubin level greater than 20.52 μmol/L were also excluded. Finally, a cross-sectional sample of 3,399 subjects was included in the analysis.

### Clinical Variables

Cigarette smoking was defined as at least one pack per month for at least half a year. Smoking habits were categorized into current smoker versus non-current smoker. Alcohol drinking was defined as at least once per week, and was further classified as current drinker versus non-current drinker. Habitual exercise was defined as a minimum of 20 minutes of exercise per time, three times or more per week. The body mass index (BMI) was calculated as an individual’s weight in kilograms divided by the square of height in meters. Blood pressure was measured in both arms using a blood pressure monitor, with subjects in a supine position and after at least 15 minutes of rest, and right brachial systolic blood pressure (SBP) and diastolic blood pressure (DBP) were used in the analysis. Pre-hypertension was defined as SBP/DBP of 120-139/80-89 mmHg and normotension as SBP/DBP < 120/80 mmHg according to the JNC-7 guidelines [17]. Mean arterial pressure (MAP) was calculated as 1/3(SBP) + 2/3(DBP). Fasting blood samples were taken in the morning following at least a 10-hour fast and abstinence from smoking for more than 24 hours. Fasting plasma glucose (FPG), glycosylated hemoglobin (HbA1c), serum total cholesterol (TC), triglyceride (TG), high density lipoprotein cholesterol (HDL-C), AST, ALT, total bilirubin, and creatinine were obtained using a clinical chemistry analyzer.

### Measurement of Arterial Stiffness

Participants were examined after at least 5 minutes of supine rest. We obtained right side brachial and ankle blood pressure and arterial pulse waves by an automatic vascular screening device (BP-20RPE, Colin Medical Technology, Komaki, Japan). Pulse waves from the brachial and tibial arteries were recorded from the sensors in pneumatic cuffs wrapped on both upper arms and ankles. BaPWV was computed automatically by dividing the transmission distance by the transit time, where the transmission distance from the upper arm to the ankle was calculated according to the individual's body height, and the transit time was defined as the interval between the rise delay of brachial and tibial waveforms. Increased arterial stiffness was defined as right side baPWV ≥1,400 cm/s [15].

### Statistical analyses

Statistical analyses were performed using SPSS software (version 17.0, SPSS, Inc., Chicago, IL). Intergroup comparisons between subjects with and without increased arterial stiffness were conducted using Pearson chi-square analysis for categorical variables and the Student’s *t*-test for continuous variables. Both multiple linear and logistic regression models were used to analyze the association between serum total bilirubin level and baPWV (continuous / ≥ 1,400 cm/s) in subjects with pre-hypertension and normotension for both genders after adjustment for age, BMI, current smoking, alcohol drinking, habitual exercise, FPG, MAP, and TC/HDL-C ratio. The dataset for the analyses is provided as S1 File. P < 0.05 was considered statistically significant.

## Results

A total of 3,399 subjects met the inclusion criteria for this study. Table 1 shows the baseline characteristics of study population. Individuals with increased arterial stiffness (baPWV ≥ 1,400 cm/s) were more likely to be older and had higher blood pressure (SBP, DBP and MAP), TC/HDL-C ratio, TG, FPG, AST and ALT, and a higher proportion of pre-hypertension in both genders. In addition, female subjects with increased arterial stiffness had a higher BMI. However, the bilirubin level was not significantly different between subjects with and without increased arterial stiffness for both genders in univariate analysis.

**Table 1:** Comparison of baseline characteristics between subjects with and without increased arterial stiffness (baPWV ≥ 1,400 cm/s) by gender.

Variables	Men (n = 1851)			Women (n = 1548)		
	Increased arterial stiffness		P value	Increased arterial stiffness		P value
	No	Yes		No	Yes	
	(n = 1354)	(n = 497)		(n = 1207)	(n = 341)	
Age (year)	42.5±10.0	53.8±10.8	<0.001	42.7±10.2	57.4±9.2	<0.001
BMI (kg/m^2^)	24.4±3.0	24.5±2.7	0.82	22.3±3.2	24.3±3.9	<0.001
Current smoking	212 (15.7)	84 (16.9)	0.52	25 (2.1)	3 (0.9)	0.15
Current alcohol use	231 (17.1)	77 (15.5)	0.42	34 (2.8)	4 (1.2)	0.08
Vigorous exercise	115 (8.5)	37 (7.4)	0.47	54 (4.5)	21 (6.2)	0.20
Systolic BP (mmHg)	115.3±9.1	123.9±8.9	<0.001	106.1±10.0	122.6±9.8	<0.001
Diastolic BP (mmHg)	69.1±7.6	76.01±6.9	<0.001	61.7±7.6	71.0±7.5	<0.001
MAP (mmHg)	99.9±8.1	107.9±7.6	<0.001	91.3±8.7	105.4±8.3	<0.001
BP status						
Normotension	899 (66.4)	156 (31.4)	<0.001	1079 (89.4)	117 (34.3)	<0.001
Pre-hypertension	455 (33.6)	341 (68.6)		128 (10.6)	224 (65.7)	
TC (mmol/L)	5.1±0.9	5.3±1.0	<0.001	5.0±0.9	5.5±1.0	<0.001
HDL-C (mmol/L)	1.2±0.3	1.2±0.3	<0.05	1.6±0.4	1.5±0.70	<0.001
TC/HDL-C ratio	4.4±1.2	4.7±1.3	<0.001	3.3±0.9	4.0±1.2	<0.001
Triglyceride (mmol/L)	1.6±1.1	1.8±1.4	<0.05	1.0±0.6	1.4±0.9	<0.001
FPG (mmol/L)	5.0±1.0	5.4±1.6	<0.001	4.7±0.7	5.3±1.8	<0.001
AST (U/L)	23.8±5.2	25.1±5.5	<0.05	21.1±5.0	23.5±5.3	<0.001
ALT (U/L)	26.9±10.3	28.6±10.6	<0.001	18.1±8.3	21.6±8.7	<0.001
Total bilirubin (μmol/L)	12.8±4.2	12.7±4.0	0.55	10.6±3.9	10.9±3.9	0.15
Creatinine (μmol/L)	84.6±10.6	84.6±11.4	0.93	60.7±8.4	61.5±8.8	0.12

Data expressed as mean ± standard deviation or number (%). Abbreviations: BMI, body mass index; BP, blood pressure; MAP, mean arterial pressure; TC, total cholesterol; HDL-C, high-density lipoprotein cholesterol; FPG, fasting plasma glucose; AST, aspartate aminotransferase; ALT, alanine aminotransferase.

Based on multiple linear regression analysis, serum bilirubin level was inversely associated with baPWV in non-hypertensive men (β = -0.066, p < 0.001) but not in non-hypertensive women (β = -0.008, p = 0.627) after adjustment for age, BMI, current smoking, alcohol drinking, habitual exercise, FPG, MAP, and TC/HDL-C ratio. In addition, the inverse relationship between bilirubin level and baPWV was found statistically significant only in pre-hypertensive men (β = -0.110, p < 0.001) in further stratified analysis (data not shown). Multiple logistic regression was used to examine the effect of serum total bilirubin level on increased arterial stiffness in subgroups with pre-hypertension and normotension, based on gender-stratified analysis (shown in Table 2). After adjustment for other variables, the results showed that total bilirubin was inversely associated with increased arterial stiffness in men with pre-hypertension (OR = 0.955, 95% CI = 0.916–0.996, p < 0.05) but not normotension. In women, the relationship between total bilirubin level and increased arterial stiffness did not reach statistical significance for both subgroups with pre-hypertension and normotension. In addition, age and MAP were independently associated with increased arterial stiffness in pre-hypertensive and normotensive subgroups for both genders. In men, current smoking was positively associated with increased stiffness, while BMI was negatively related to increased arterial stiffness in both pre-hypertensive and normotensive subgroups.

**Table 2:** Multiple logistic regression analysis for the relationship between serum total bilirubin and increased arterial stiffness in subjects with pre-hypertension or normotension by gender.

Variables	Men		Women	
	Pre-hypertension	Normotension	Pre-hypertension	Normotension
	(n = 796)	(n = 1055)	(n = 352)	(n = 1196)
	OR (95%CI)	OR (95%CI)	OR (95%CI)	OR (95%CI)
Age (year)	1.101 (1.081–1.122)***	1.119 (1.095–1.144)***	1.129 (1.093–1.167)***	1.143 (1.111–1.177)***
BMI (kg/m^2^)	0.892 (0.836–0.952)**	0.863 (0.793–0.938)**	0.984 (0.917–1.057)	0.981 (0.906–1.062)
Current smoking	1.908 (1.128–3.227)*	1.963 (1.164–3.311)*	6.822 (0.480–96.947)	0.000
Current alcohol use	0.609 (0.368–1.008)	1.381 (0.821–2.323)	0.719 (0.086–5.981)	1.200 (0.247–5.824)
Vigorous exercise	0.999 (0.537–1.858)	0.516 (0.238–1.118)	1.619 (0.579–4.526)	0.650 (0.201–2.100)
FPG (mmol/L)	1.025 (0.910–1.154)	1.122 (0.963–1.308)	1.025 (0.848–1.239)	1.203 (0.913–1.585)
Mean arterial pressure (mmHg)	1.151 (1.108–1.195)***	1.162 (1.109–1.218)***	1.102 (1.041–1.167)**	1.161 (1.113–1.212)***
TC/HDL-C ratio	1.067 (0.929–1.226)	1.394 (1.179–1.647)***	1.165 (0.902–1.505)	1.008 (0.793–1.280)
Total bilirubin (μmol/L)	0.955 (0.916–0.996)*	0.998 (0.951–1.048)	1.042 (0.975–1.114)	0.969 (0.912–1.029)

*p<0.05,

**p<0.01,

***p<0.001

Abbreviations: BMI, body mass index; FPG, fasting plasma glucose; TC/HDL-C, total cholesterol/high-density lipoprotein cholesterol.

## Discussion

Based on the different status of blood pressure, Zhang et al. demonstrated an independent inverse relationship between bilirubin level and baPWV in hypertensive men [14]. As for non-hypertensive subjects, our study showed an inverse relationship between bilirubin level and baPWV in men, which is compatible with the findings in men without a history of hypertension by Li et al [13], while Zhang et al. failed to replicate this finding [14]. The heterogeneity of the non-hypertensive population, which includes pre-hypertensive and normotensive subjects, may be one possible explanation for the discrepancy between these works. Therefore, we further divided the non-hypertensive group into pre-hypertension and normotension. To the best of our knowledge, this study is the first one showing an inverse association between bilirubin level and increased arterial stiffness in men with pre-hypertension but not normotension. However, neither pre-hypertensive nor normotensive women exhibited a negative association between bilirubin level and increased arterial stiffness. This result is consistent with the findings in non-hypertensive women in the studies by Li et al. [13] and Zhang et al [14].

This study found an inverse relationship between bilirubin level and increased arterial stiffness in pre-hypertensive subjects, and this finding is consistent with the beneficial effect of bilirubin on other surrogates of cardiovascular risk. Studies have shown that subjects with lower serum bilirubin concentrations exhibit significant endothelial dysfunction, increased carotid intima-media thickness, and higher coronary artery calcification [18–20], which are predictors for atherosclerosis. The protective effect of bilirubin on cardiovascular risk seems to be related to its role as an endogenous antioxidant. As compared to normotensive subjects, pre-hypertensive ones had higher oxidative stress [21]. In order to counteract the harmful effects of oxidative stress, endogenous antioxidants are the first line of defense to alleviate oxidative damage [22]. Therefore, an increased bilirubin level to protect against the risk of cardiovascular diseases may occur in pre-hypertensive subjects, but not normotensive ones. However, the exact mechanism underlying this needs further research.

Sex hormones are known to be intimately involved in the pathogenesis of atherosclerosis. This study failed to detect a significantly protective effect of bilirubin against arterial stiffness in women, and this is in line with previous studies [13,14]. The lower serum bilirubin in women may be due to the influence of estrogen [23], possibly via induction of hepatic UDP-glucuronosyltransferase, resulting in increased bilirubin excretion [24]. Furthermore, estrogen has been demonstrated to inhibit atherosclerotic plaque development and progression, and appears to have a beneficial effect on atherosclerosis and vessel wall physiology [25]. To our knowledge, scarce studies focused on the effects of estrogen on arterial stiffness, and one study demonstrated a high content of estrogen receptor α (ERα) correlates with a lower collagen concentration, indicating that estrogen protects against vascular collagen accumulation making the vessel more distensible through activation of ERα [26]. In addition, estrogen may also mediate a variety of chronic inflammatory diseases, including cardiovascular diseases, by inhibiting the expression of tumor necrosis factor [27]. The beneficial effects of estrogen may thus outweigh the protective effect of bilirubin against arterial stiffness in women.

This study found that current smoking was significantly associated with increased arterial stiffness in both pre-hypertensive and normotensive men, but not in women. Since only 28 female subjects reported current smoking, the insignificant association between smoking and arterial stiffness in women might not have been detected in such a small group. The association between BMI and arterial stiffness remains unsettled. Some studies indicate that BMI is positively related to arterial stiffness [28,29], while others and the current study report a negative association between them [30–32]. The influence of obesity on baPWV might be mediated by the intermediate variables of other cardiovascular risk factors. However, further studies may be needed to investigate the effect of obesity on arterial stiffness.

This study has some limitations. First, we measured blood pressure with participants in the supine position. According to the recommendation statement for blood pressure measurement by American Heart Association, it is widely accepted that diastolic pressure measured while sitting is higher by 5 mm Hg than when measured supine, while there is less agreement about systolic pressure [33]. Based on a lower supine diastolic blood pressure, the prevalence of pre-hypertension could be underestimated, which may result in a lower statistical power to detect the association between bilirubin level and baPWV. However, our main finding still showed a significant inverse association between serum bilirubin level and arterial stiffness in pre-hypertensive men. Second, the cross-sectional design makes it difficult to determine the causal or temporal relationship between bilirubin and arterial stiffness, as shown by baPWV. Third, cfPWV is considered the current gold standard of arterial stiffness measurement. However, baPWV has been shown to be associated with cardiovascular risk factors in a manner similar to cfPWV [34]. Recent longitudinal studies also demonstrate that baPWV is a significant predictor of cardiovascular events and mortality among high-risk patients and the general population [7,34]. Therefore, baPWV may be another surrogate index for cardiovascular diseases. Finally, the study subjects were confined to a Taiwanese population, and thus the findings may not be generalizable to other ethnic groups.

In conclusion, this study revealed that serum bilirubin level was inversely associated with increased arterial stiffness in men with pre-hypertension, but not normotension. The association between bilirubin level and increased arterial stiffness was not significant in women. Because recent evidence have indicated that pre-hypertension, even in the low-range pre-hypertension (120–129/80–84 mmHg), elevates the risk of cardiovascular diseases [35,36], it is important to identify the subgroup of pre-hypertension, who has a higher risk for cardiovascular events, and to encourage early interventions. Although further studies are warranted, this study provides directions for further research on the potential protective effects against cardiovascular diseases of bilirubin.

## Supporting Information

S1 File Dataset for the final analyses. (CSV)
